# Supplementary material for: A bifunctional asparaginyl endopeptidase efficiently catalyzes both cleavage and cyclization of cyclic trypsin inhibitors
Source: Nat Commun. 2020 Mar 27;11:1575. doi: 10.1038/s41467-020-15418-2 (PMC7101308; doi:10.1038/s41467-020-15418-2)
Supplement: Supplementary file 1 — Supplementary Information [file 41467_2020_15418_MOESM1_ESM.pdf]

Supplementary Information

A bi-functional asparaginyl endopeptidase efficiently catalyzes both cleavage and cyclization of cyclic trypsin inhibitors

Du et al.

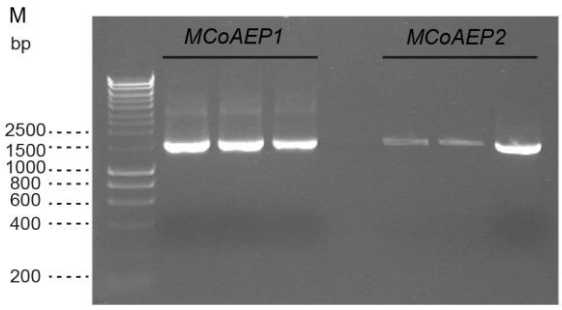

Supplementary Figure 1. Cloning of MCoAEPs. PCRs were performed with three different cDNA templates and the following primers derived from RNAseq transcriptome data: *MCoAEP1* Forward: 5'- GGG GAC AAG TTT GTA CAA AAA AGC AGG CTA TGA CTC GTA TCC CCA ACG GAG -3', Reverse: 5'- GGG GAC CAC TTT GTA CAA GAA AGC TGG GTT CAA GCA GTG AAG CCC TTG TGC -3' and *MCoAEP2* Forward: 5'-GGG GAC AAG TTT GTA CAA AAA AGC AGG CTA TGG CCG CCC TGA ACT CTG-3', Reverse: 5'-GGG GAC CAC TTT GTA CAA GAA AGC TGG GTT CAA GCA CTA AAA CCA CCA CCT TCA TGC-3'. M: DNA Hyperladder 1. The size of the PCR products is in agreement with the typical size of AEP cDNAs (~1.5 kbp).

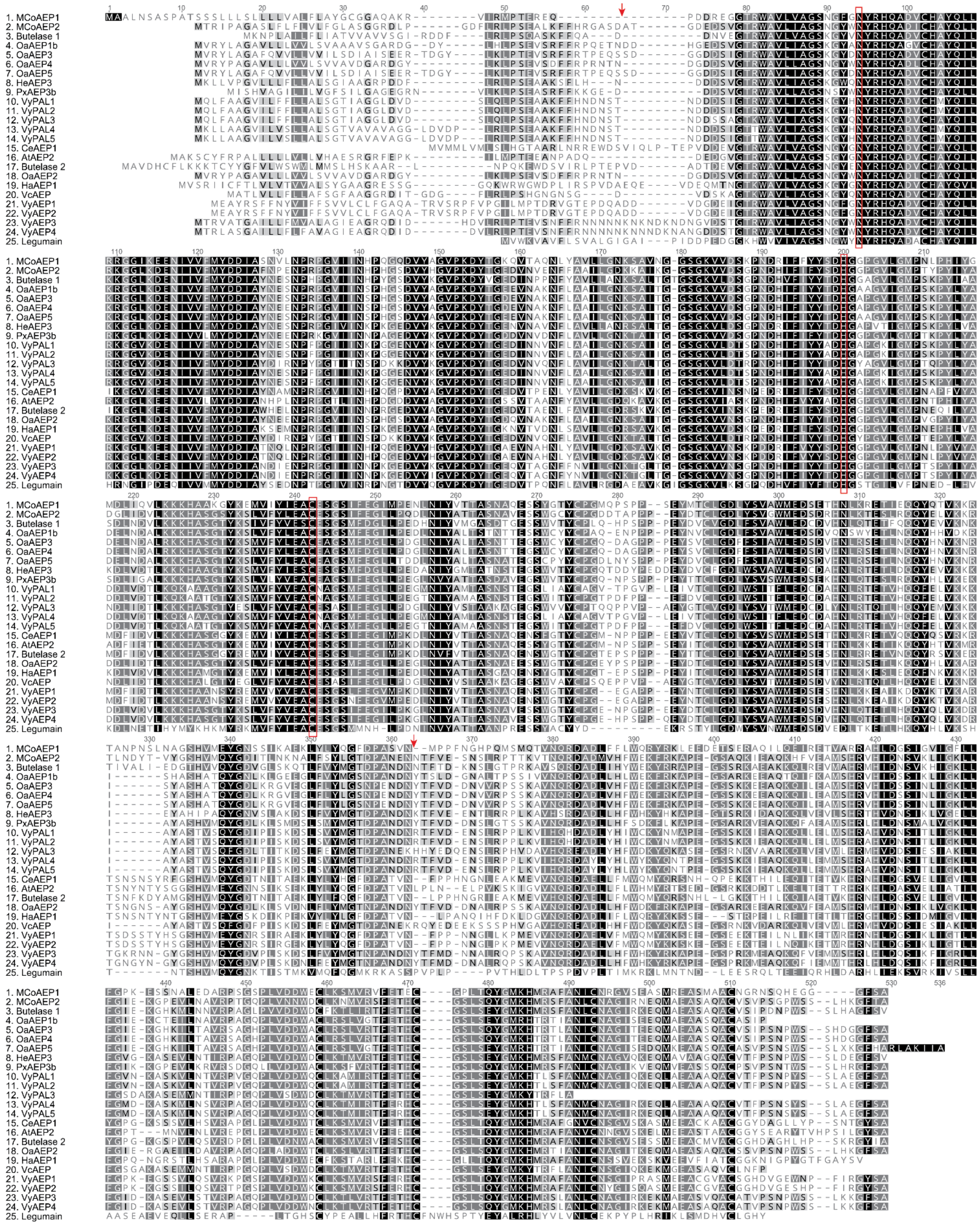

Supplementary Figure 2. Protein sequence alignment of AEP homologues. The identified N- and C-terminal auto-processing sites of MCoAEP2 are indicated with arrows. The residues highlighted with red boxes form the conserved catalytic triad (Asn, His, Cys).

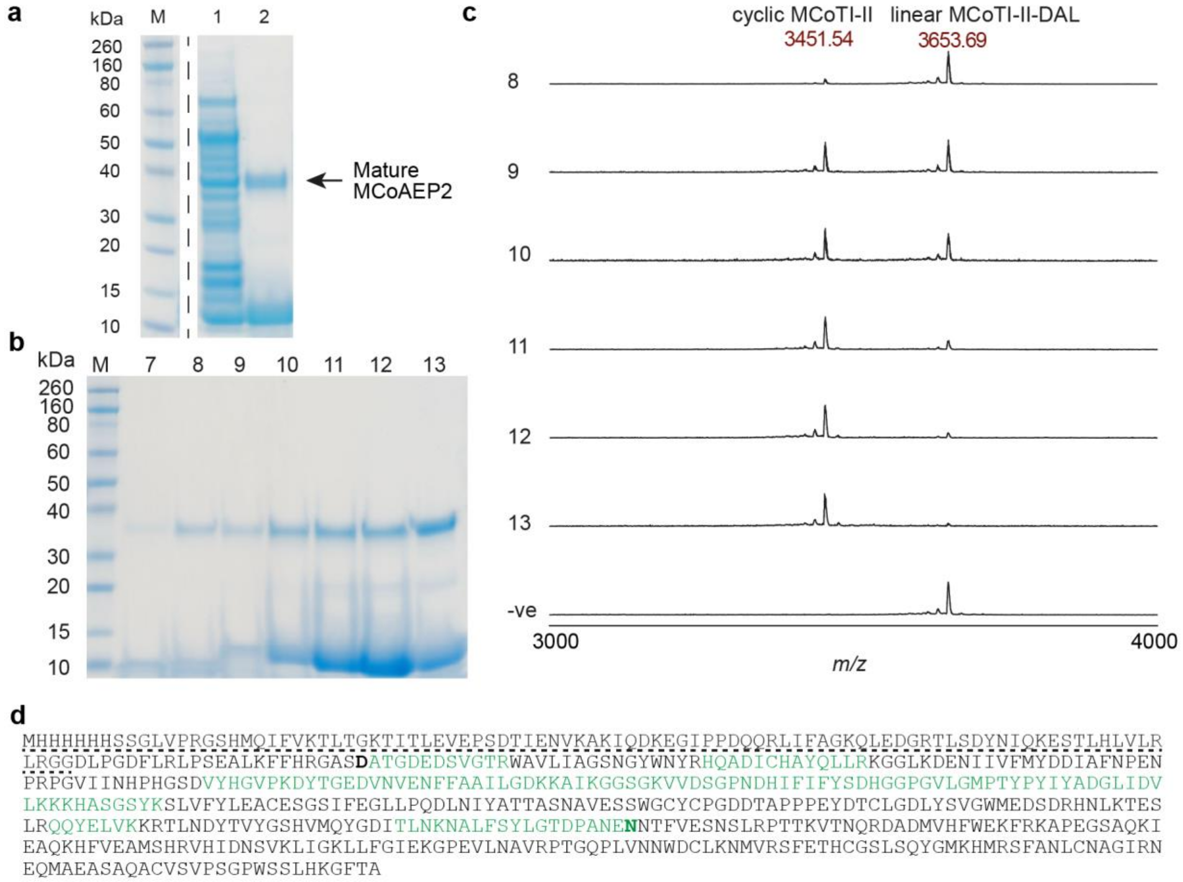

Supplementary Figure 3. Recombinant expression of MCoAEP2 in *E. coli*. SDS-PAGE protein bands were visualized by staining with InstantBlue™ Coomassie. Recombinant expression of MCoAEP2 was performed independently three times and a representative MCoAEP2 expression experiment is shown here. **a** Activation of MCoAEP2. M: protein ladder, lane1: crude IMAC-purified MCoAEP2 sample and (lane 2) after activation at pH 4, 37°C for 30 min. **b** Cation exchange chromatography fractions (7 to 13) of activated MCoAEP2. **c** Enzymatic cyclization assay for monitoring MCoAEP2 activity. Assays were conducted at pH 5 and 22°C, and analyzed by MALDI-TOF at t = 30 min; -ve: control reaction without MCoAEP2. **d** Sequence coverage observed following nanoLC-MS/MS analysis of a tryptic digest of the active band of mature MCoAEP2 (panel **a,** lane 2) derived from recombinant His6-ubiquitin-MCoAEP2 fusion protein expressed in *E .coli*. Sequence with dashed underline denotes the fusion protein portion, and green text indicates peptides assigned with > 95% confidence in ProteinPilot (ProteinPilot™ Software 4.0). Bold residues Asp and Asn are indicated as auto-activation sites.

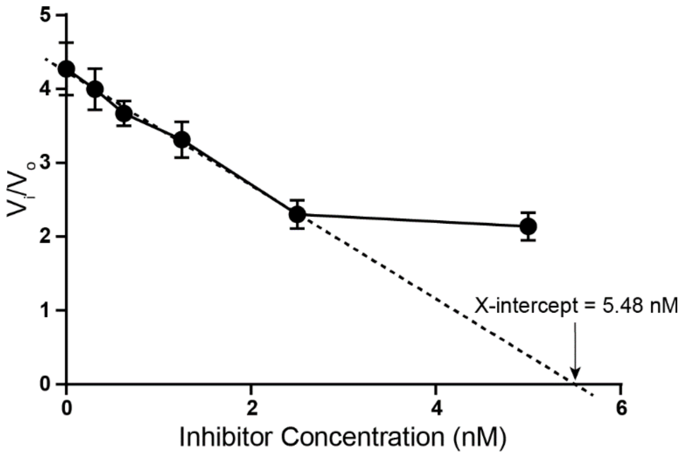

Supplementary Figure 4. Active site titration of activated MCoAEP2 with Ac-YVAD-CMK. The X-axis intercept corresponds to the active site concentration of MCoAEP2 in the reaction, which was determined to be 5.48 nM (95% Confidence Intervals: 4.998–6.083). V_i: absorbance change per minute; V_o: absorbance from substrate only control group. The assay was done with three biological replicates. Data are presented as means ± SEM (n = 3).

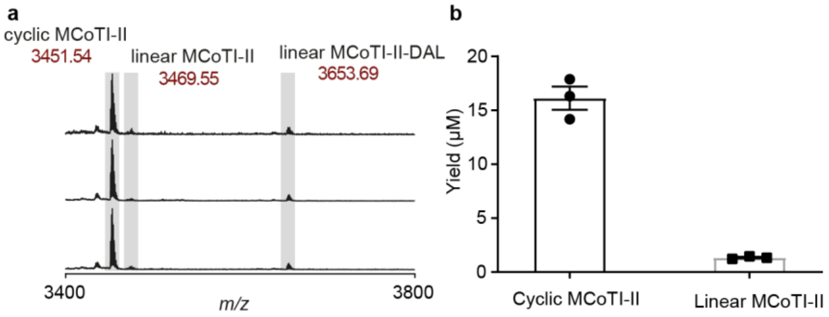

Supplementary Figure 5. Qualitative and quantitative analysis of products produced from incubation of MCoAEP2 (75 nM) with MCoTI-II-DAL substrate (25 µM) at 22°C for 20 h. **a** MALDI-MS analysis of 3 biological replicates. **b** QTRAP quantification of the same reactions. Data are presented as means ± SEM (n = 3).

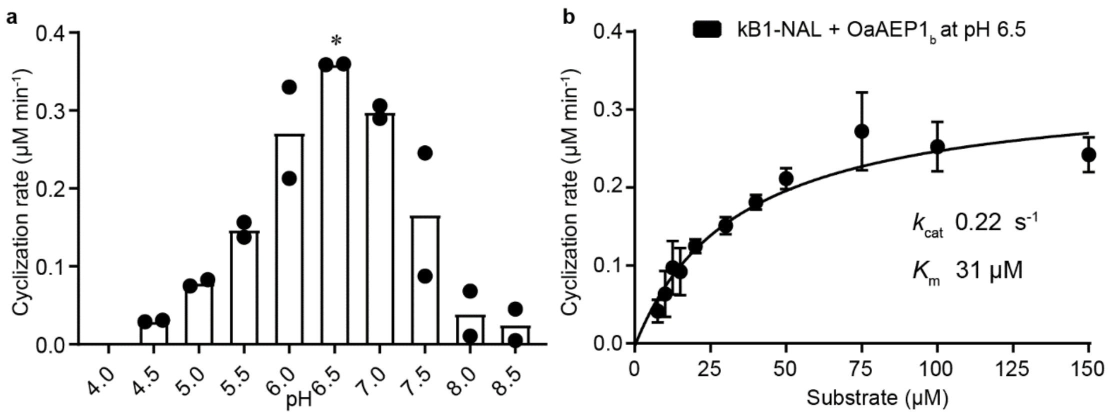

Supplementary Figure 6. pH preference and Michaelis-Menten parameters for OaAEP1$_b$-mediated cyclization of kB1-NAL. **a** pH preference of OaAEP1$_b$ cyclization activity using kB1-NAL as a substrate. Experiments were conducted in duplicate (n = 2) at 22ºC for 5 min in the presence of 25 nM of OaAEP1$_b$ and 50 µM kB1-NAL at varying pH. **b** Michaelis-Menten plot of OaAEP1$_b$ with kB1-NAL substrate. Kinetic assays were performed with 25 nM of OaAEP1$_b$ with varying kB1-NAL substrate concentrations (µM) at optimal pH (6.5; indicated with an asterisk in panel **a**). Experiments were performed in triplicate (n = 3) and data are presented as means ± SEM.

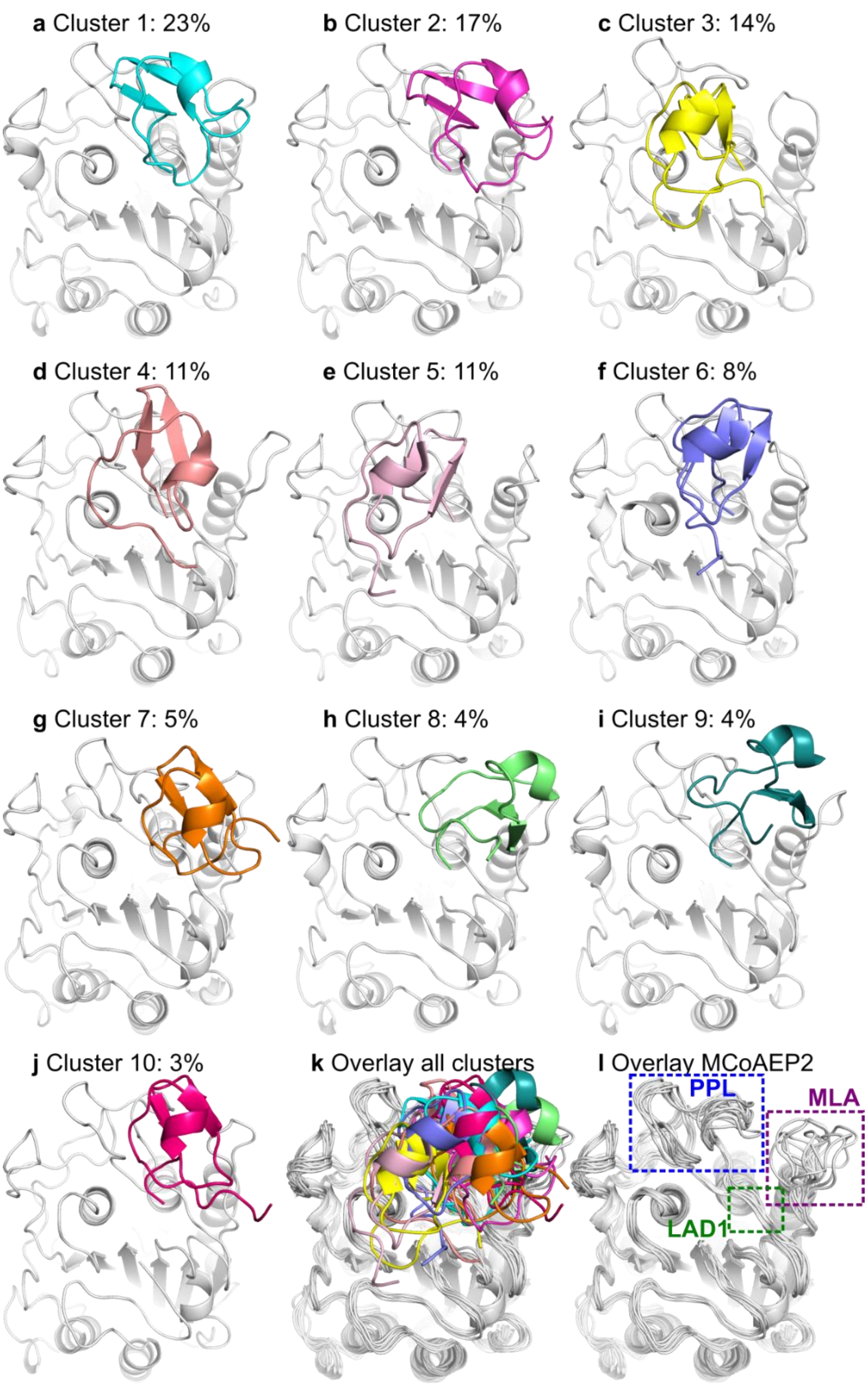

Supplementary Figure 7. Binding modes observed during the molecular dynamics simulation of MCoAEP2 covalently linked to an intermediate form of MCoTI-II substrate. All the frames from the 1 µs molecular dynamics simulation of the complex between MCoTI-II and MCoAEP2 were grouped in 10 clusters using a k-mean. The individual centroid frames from these 10 clusters are shown in panels **a** to **j**, and an overlay of all the centroid frames is shown in panel **k** and **l**. MCoAEP2 is shown in white, and MCoTI-II is shown in different colors in each centroid frame. The percentage of the total number of frames in each cluster is indicated in panels **a** to **j**. Panel **l** shows the same overlay of MCoAEP2 as in panel **k** but MCoTI-II was not represented in panel **l** to illustrate the conformational flexibility of the enzyme. The PPL, MLA and LAD1 loops are represented in panel **l**.

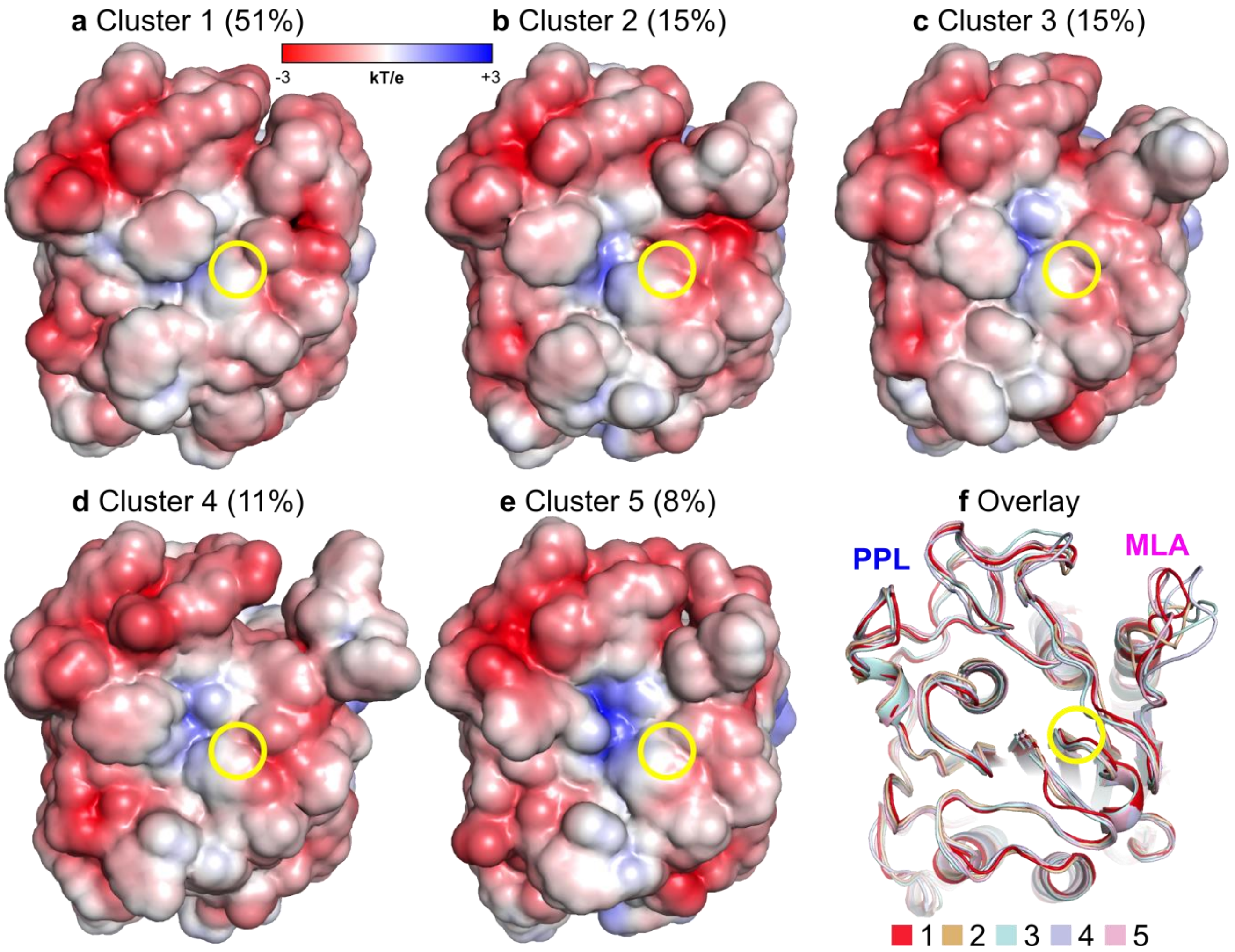

Supplementary Figure 8. Conformation and electrostatic potential of the centroid frames resulting from a k-mean analysis of the 1 µs molecular dynamics simulation of the MCoAEP2 apo state. The percentage of the total number of frames in each cluster is indicated between parentheses in panels **a** to **e**. Panel **f** shows an overlay of the backbone of the centroid frames displayed in panels **a** to **e**. In panels **a** to **e**, the electrostatic potential computed with APBS 1.4 was mapped on the solvent accessible surface and colored from -3 kT/e (red) to + 3 kT/e (blue). In panel **f**, the centroid frames corresponding to each cluster are colored differently from each other as indicated in the legend. The MLA and PPL loops are also indicated on panel **f**, suggesting that the MLA loop is flexible. The Asx binding pocket is indicated by a yellow circle in all panels.

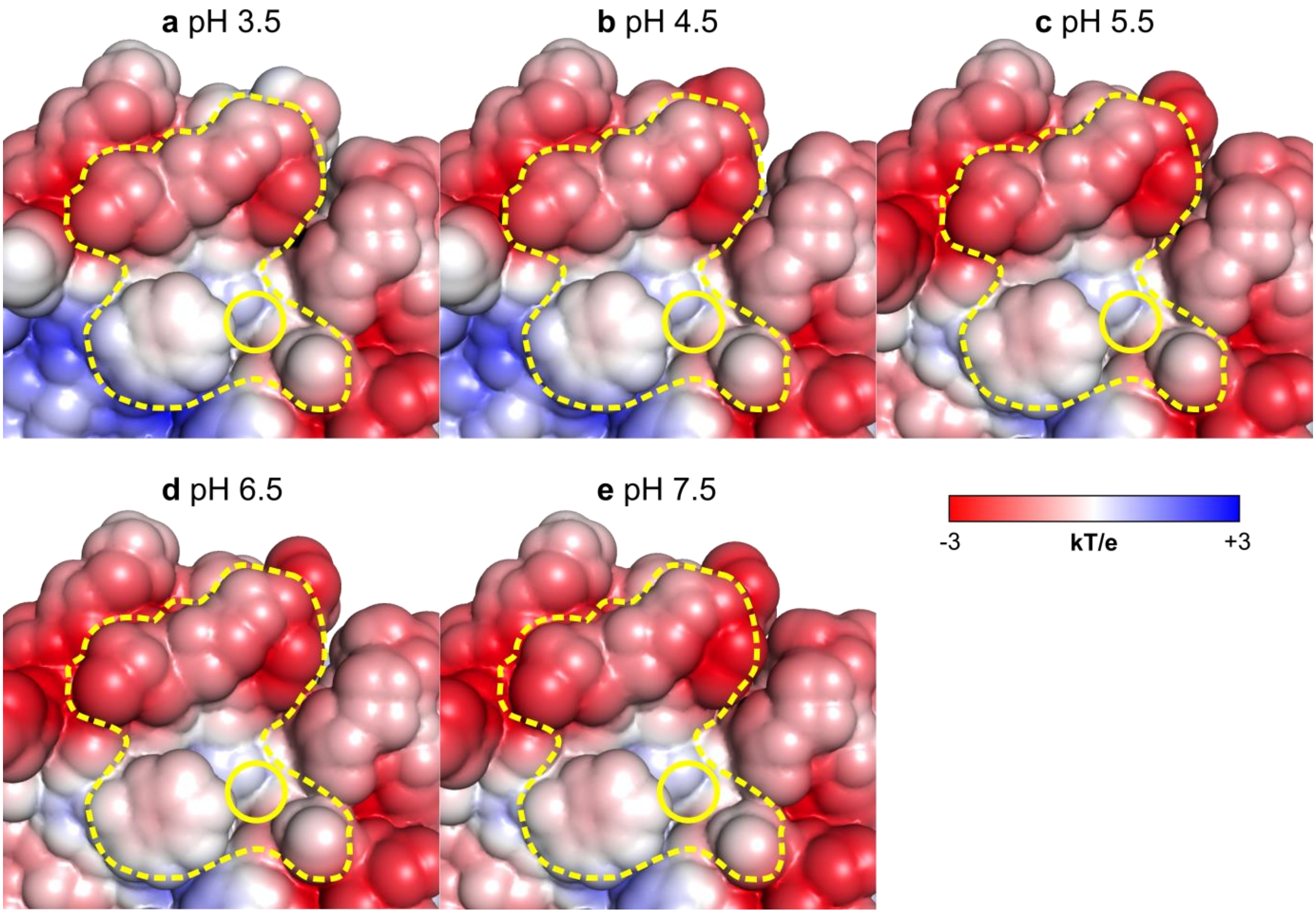

Supplementary Figure 9. Assuming that the catalytic Cys218 is deprotonated, the electrostatic potential of MCoTI-II binding site of MCoAEP2 is similar at all studied pH. The electrostatic potential was computed with APBS 1.4 at various pH (**a**: pH 3.5; **b**: pH 4.5; **c**: pH 5.5; **d**: pH 6.5; **e**: pH 7.5) and mapped on the solvent accessible surface, which was colored from -3 kT/e (red) to + 3 kT/e (blue). The binding site of MCoTI-II, which was identified using simulation of the MCoTI-II/MCoAEP2 complex, is delimited by a yellow dashed line. The Asx binding pocket is indicated by a yellow circle in all panels.

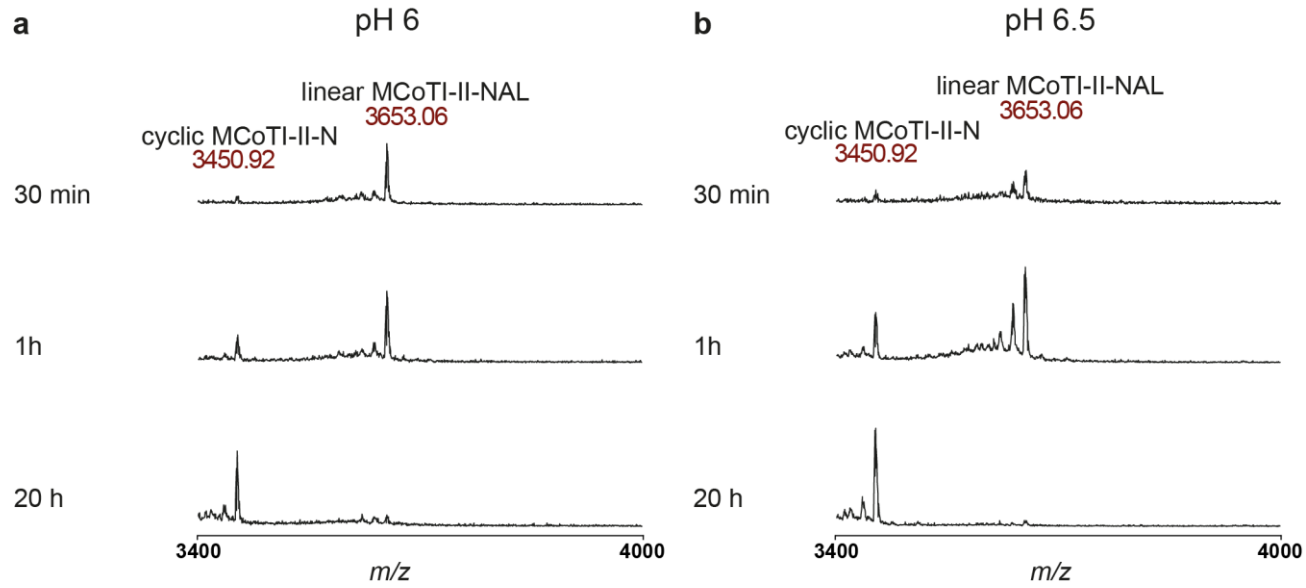

Supplementary Figure 10. Processing of MCoTI-II-NAL by OaAEP1$_b$. Reactions were carried out with 1 µM OaAEP1$_b$ and 50 µM substrate and analyzed by MALDI-MS at the indicated time points. **a** pH 6.0 or **b** pH 6.5. Observed monoisotopic masses ($[M+H]^+$) are indicated.

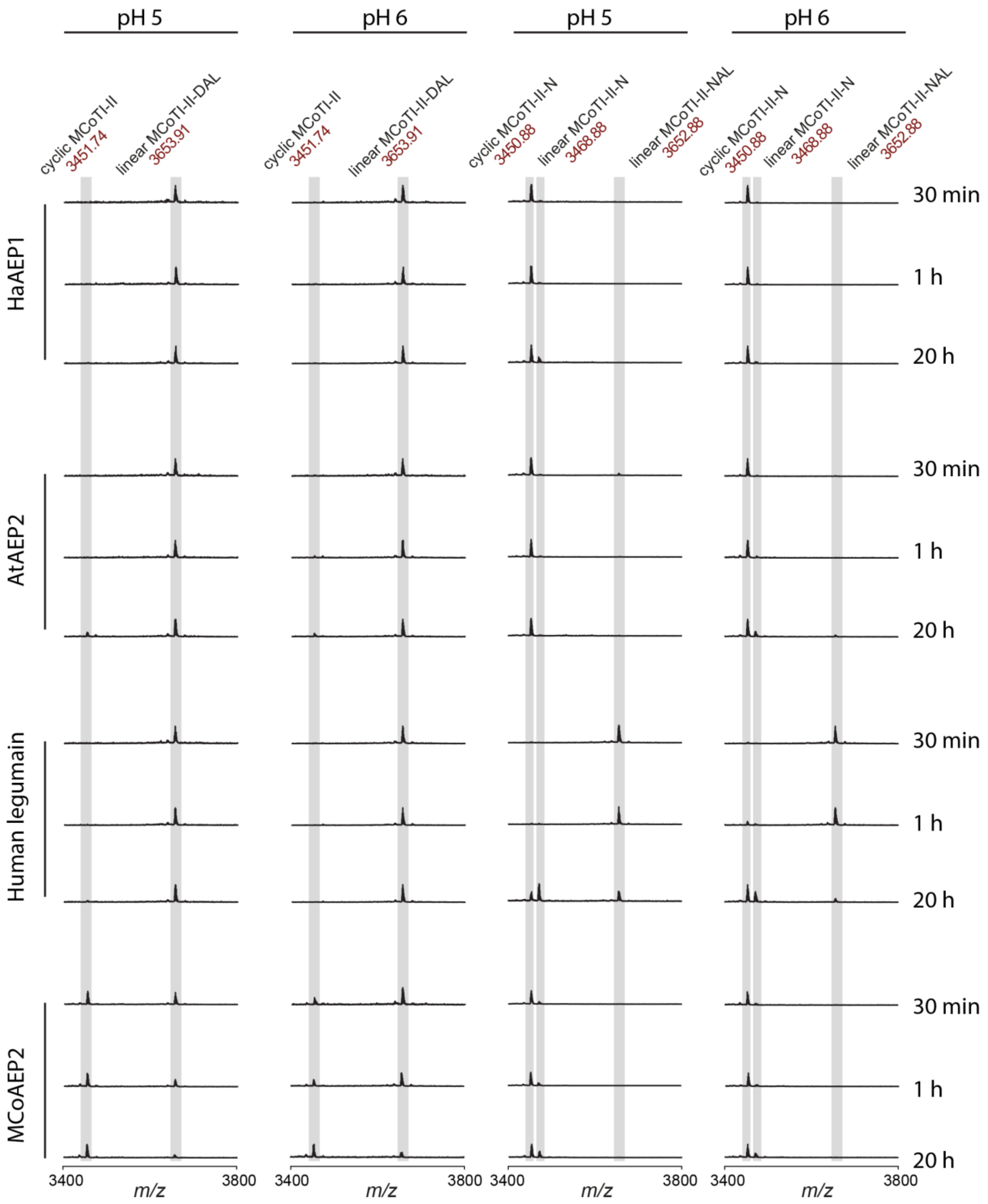

Supplementary Figure 11. Activity of a panel of recombinant AEPs against MCoTI-II-DAL and MCoTI-II-NAL substrates (50 µM) at pH 5 or 6 at 22ºC analyzed after 30 min, 1h, and 20 h by MALDI-MS. HaAEP1 (*Helianthus annuus*), AtAEP2 (*Arabidopsis thaliana*) and Human legumain were quantified by absorption at 280 nm and added to the assay buffer to a final concentration of 0.1 mg/ml, 0.04 mg/ml and 0.1 mg/ml, respectively. MCoAEP2 concentration was 50 nM as determined by active site titration.

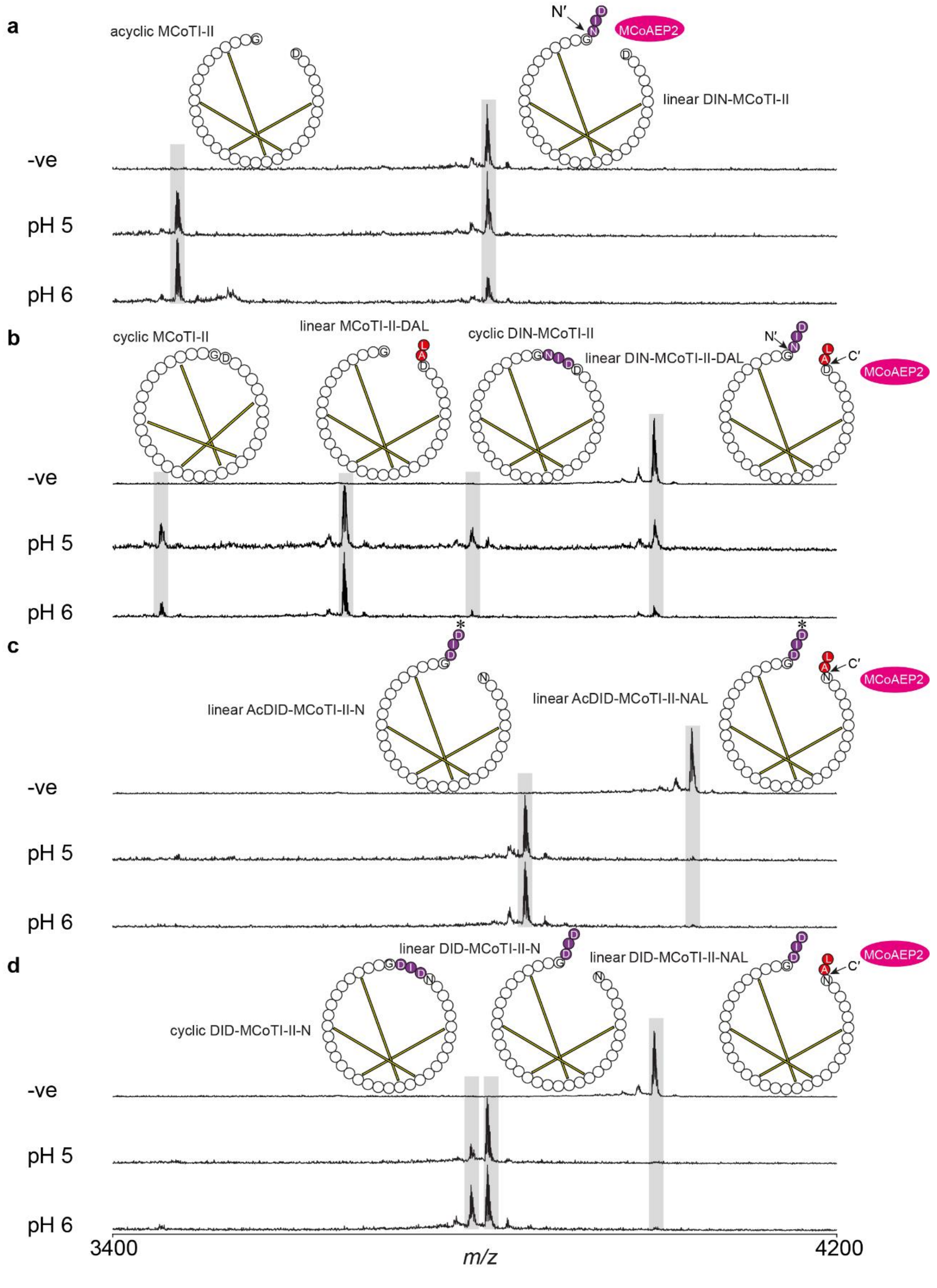

Supplementary Figure 12. N- and C-terminal processing of synthetic MCoTI substrate precursors by MCoAEP2. **a** DIN-MCoTI-II, **b** DIN-MCoTI-II-DAL, **c** AcDID-MCoTI-II-NAL (acetylated N-terminus is indicated by an asterisk) or **d** DID-MCoTI-II-NAL at 50 µM substrate concentration were incubated with 50 nM MCoAEP2 at 22 ºC at pH 5 or pH 6 for 30 min. Reactions were analyzed by MALDI-MS and a no enzyme control is shown for comparison (-ve). N′ and C′ indicate N- and C-terminal processing sites and leader and follower peptides are shown in purple and red, respectively.

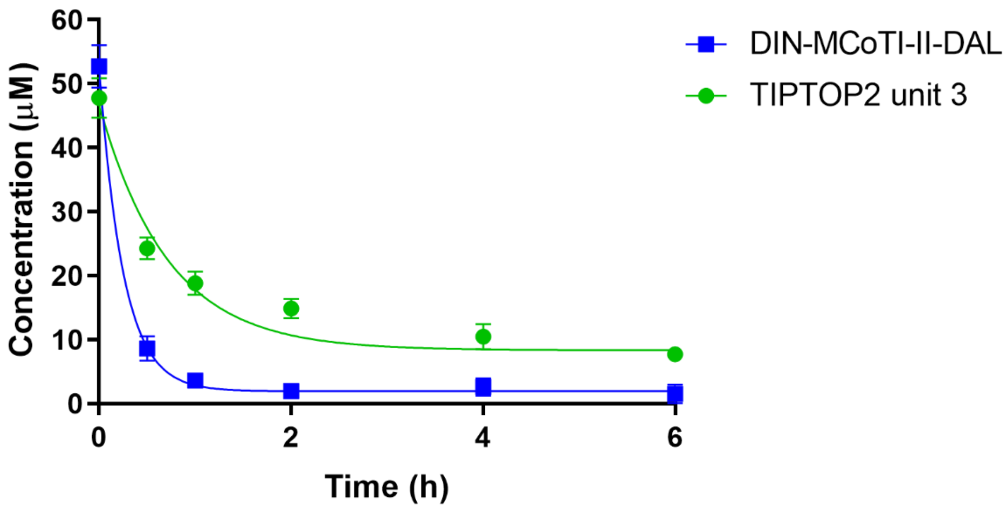

Supplementary Figure 13. Processing of full-length MCoTI precursor (TIPTOP2 unit 3) and truncated DIN-MCoTI-II-DAL by MCoAEP2. The substrates (50 μM) were incubated with 50 nM MCoAEP2 and substrate depletion was monitored over time via quantitative QTRAP-MS. Experiments were performed in triplicate and data are presented as means ± SEM (n = 3).

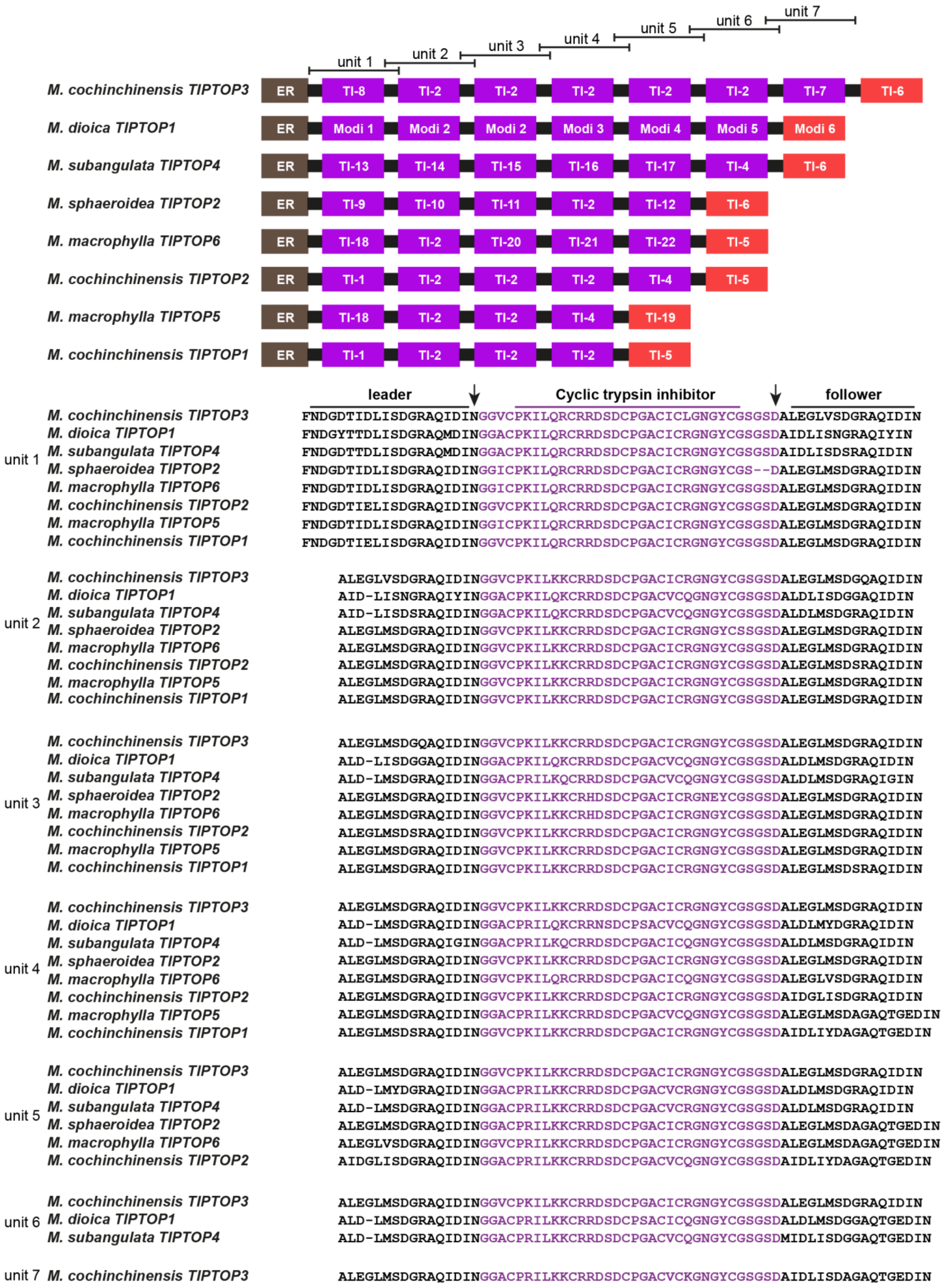

Supplementary Figure 14. *TIPTOP* gene precursor alignments from reported *Momordica* subspecies[1-3]. Cyclic trypsin inhibitor domains are highlighted in purple. Arrows indicate the N-and C-terminal processing sites.

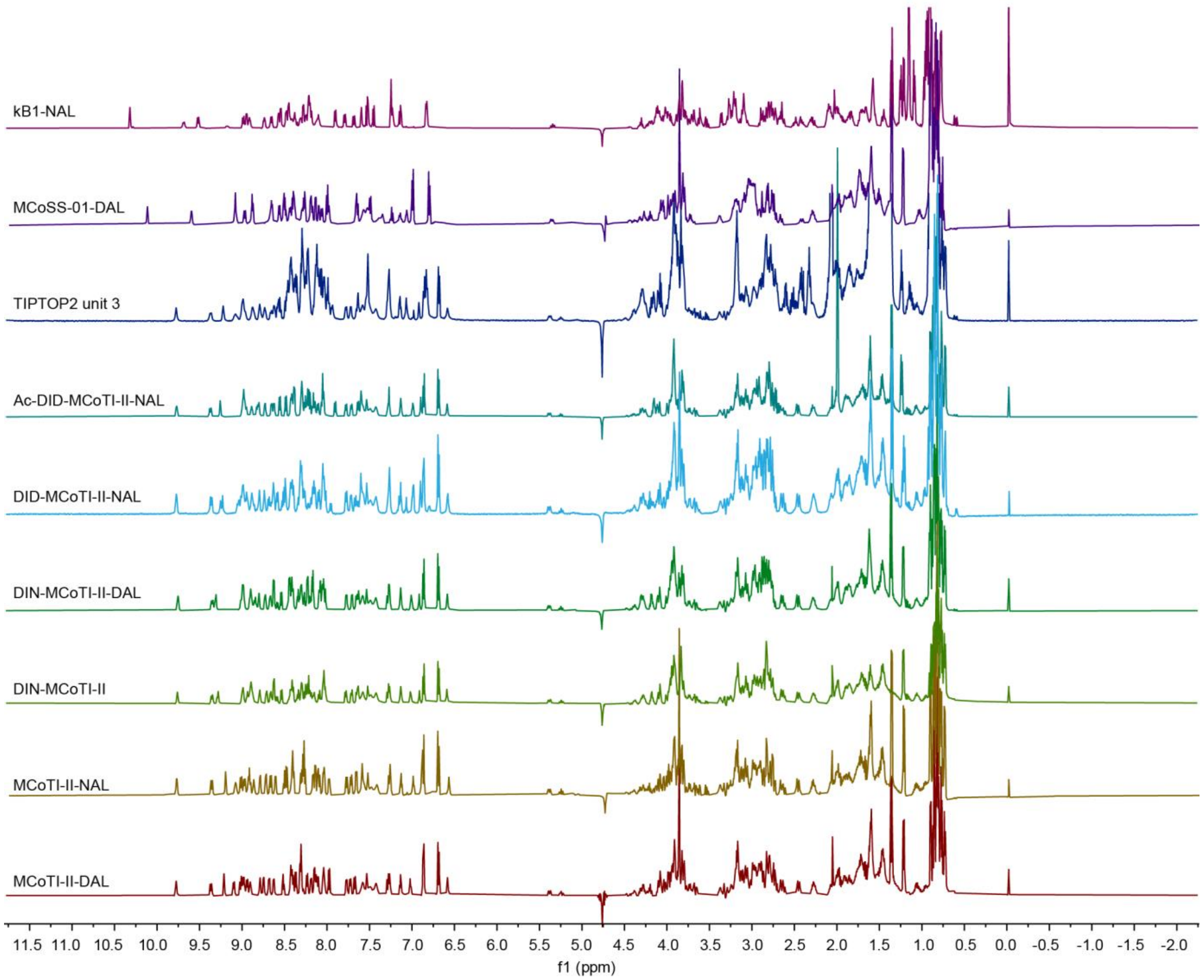

Supplementary Figure 15. 1D ^{1}H NMR spectra of peptide substrates used in this study.

Supplementary References

1. Mylne JS, *et al.* Cyclic peptides arising by evolutionary parallelism via asparaginyl-endopeptidase-mediated biosynthesis. *Plant Cell* **24**, 2765-2778 (2012).
2. Mahatmanto T, *et al.* The evolution of Momordica cyclic peptides. *Mol. Biol. Evol.* **32**, 392-405 (2015).
3. Du J, Chan LY, Poth AG, Craik DJ. Discovery and characterization of cyclic and acyclic trypsin inhibitors from Momordica dioica. *J. Nat. Prod.* **82**, 293-300 (2019).
