# Supplementary material for: A bifunctional asparaginyl endopeptidase efficiently catalyzes both cleavage and cyclization of cyclic trypsin inhibitors
Source: Nat Commun. 2020 Mar 27;11:1575. doi: 10.1038/s41467-020-15418-2 (PMC7101308; doi:10.1038/s41467-020-15418-2)
Supplement: Supplementary file 3 — Reporting Summary [file 41467_2020_15418_MOESM3_ESM.pdf]

nature research

Corresponding author(s): Dr David Craik / Dr Thomas Durek

Last updated by author(s): 27/02/2020

Reporting Summary

Nature Research wishes to improve the reproducibility of the work that we publish. This form provides structure for consistency and transparency in reporting. For further information on Nature Research policies, seeAuthors & Referees and theEditorial Policy Checklist .

Statistics

For all statistical analyses, confirm that the following items are present in the figure legend, table legend, main text, or Methods section.

n/a	Confirmed	
☐	☒	The exact sample size (n) for each experimental group/condition, given as a discrete number and unit of measurement
☐	☒	A statement on whether measurements were taken from distinct samples or whether the same sample was measured repeatedly
☒	☐	The statistical test(s) used AND whether they are one- or two-sided<br>*Only common tests should be described solely by name; describe more complex techniques in the Methods section.*
☒	☐	A description of all covariates tested
☒	☐	A description of any assumptions or corrections, such as tests of normality and adjustment for multiple comparisons
☐	☒	A full description of the statistical parameters including central tendency (e.g. means) or other basic estimates (e.g. regression coefficient) AND variation (e.g. standard deviation) or associated estimates of uncertainty (e.g. confidence intervals)
☒	☐	For null hypothesis testing, the test statistic (e.g. F, t, r) with confidence intervals, effect sizes, degrees of freedom and P value noted<br>*Give P values as exact values whenever suitable.*
☒	☐	For Bayesian analysis, information on the choice of priors and Markov chain Monte Carlo settings
☒	☐	For hierarchical and complex designs, identification of the appropriate level for tests and full reporting of outcomes
☒	☐	Estimates of effect sizes (e.g. Cohen's d, Pearson's r), indicating how they were calculated

Our web collection on statistics for biologists contains articles on many of the points above.

Software and code

Policy information about availability of computer code

Data collection	Standard Illumina platform software was used to collect RNA-seq data with the HiSeq2500 pipeline.<br>Analyst v1.6.3 (ABSciex) was used to acquire quantitative MS data for enzyme kinetics. Standard Bruker NMR software (Topspin v3.6.1) was used to acquire NMR data.
Data analysis	All software used to handle RNA-seq reads and assemble transcriptomes are publicly accessible through portals such as GitHub and SourceForge. Namely, Trinity v 2.4.0 and Trimmomatic v0.36 were used for this. References for these software packages are included in the methods. Multiquant 3.02 (ABSciex) was used to analyze quantitative MS data and Proteinpilot V4.0 was used to analyse tandem MS data. Graphpad Prism 7 was used to analyse enyzme kinetics data. CCPNMR Analysis 2.4.1 was used to assign NMR data. The three-dimensional structure of the catalytic domain of MCoAEP2 was modelled by homology using Modeller 9v21 with the HaAEP1 crystal structure as a template (PDB: 6azt). The protonation state of side chains at pH 5.5 was predicted using Propka 3.1. Electrostatic potential generated by MCoAEP2 was computed using APBS 1.4. Molecular dynamics simulations were carried out using the Amber 18 software package. All software used in this study is referenced in the main manuscript.

For manuscripts utilizing custom algorithms or software that are central to the research but not yet described in published literature, software must be made available to editors/reviewers. We strongly encourage code deposition in a community repository (e.g. GitHub). See the Nature Research guidelines for submitting code & software for further information.

Data

Policy information about availability of data

All manuscripts must include a data availability statement. This statement should provide the following information, where applicable:

- Accession codes, unique identifiers, or web links for publicly available datasets
- A list of figures that have associated raw data
- A description of any restrictions on data availability

RNA-seq data have been deposited in the NCBI-SRA database under the accession code PRJNA531039. GenBank accessions for AEPs gene sequences are as follows:

MCoAEP1 (MK770254) and MCoAEP2 (MK770255). The forcefield parameters, simulation protocol and simulation frames were submitted to Zenodo public data repository with the DOI 10.5281/zenodo.3621201. All other data supporting the findings of this study are included in the manuscript or supporting information files.

Field-specific reporting

Please select the one below that is the best fit for your research. If you are not sure, read the appropriate sections before making your selection.

☒ Life sciences ☐ Behavioural & social sciences ☐ Ecological, evolutionary & environmental sciences

For a reference copy of the document with all sections, see nature.com/documents/nr-reporting-summary-flat.pdf

Life sciences study design

All studies must disclose on these points even when the disclosure is negative.

Sample size	No statistical methods were used to pre-determine sample size.<br>1) For RNA-seq experiments all tissues sampled were sampled as single biological replicate. The aim was to capture the full diversity of sequences expressed, not to measure expression differences between tissues sampled. Thus, the single sample design is adequate for claims made. The RNAseq results, i.e. sequences of novel AEPs reported in our paper, have been independently confirmed via targeted PCR and Sanger sequencing (SI Fig.1).<br>2) For enzyme kinetics experiment, all samples were tested in 3 biological replicates. The only exception is for experiments shown in SI Fig. 6, for which biological replicates is n=2. Each biological data point (typically enzyme rates, i.e. uM product formed per minute) is the result of 4 separate measurements at defined time intervals to determine enzyme rates. The addition of an internal standard in all experiments was used to account for instrumental and technical variation. The number of biological replicates chosen were determined in pilot experiments and found to be sufficient to replicate literature values and support the enzyme kinetic claims made here.
Data exclusions	1) Some of the reads and fractions of read in the RNA-seq data have been excluded because they were filtered out due to low Phred quality scores (<Q30) and similarity to sequencing barcodes and adapters. This is standard practice for robust RNA-seq data analysis.<br>2) For enzyme kinetics experiments, no data was excluded.
Replication	Nucleotide sequences from a single RNAseq experiment were independently confirmed via targeted PCR and Sanger sequencing. For enzyme kinetics experiment, all samples were tested in 3 biological replicates (except for SI Fig.6, where n=2).
Randomization	No specific attempts to randomize samples were made and no analysis of covariance was performed.
Blinding	RNAseq data were acquired by an external contractor, the Australian Genome Research Facility.<br>For enzyme kinetic measurements, the researcher acquiring the quantitative MS data was blinded.

Reporting for specific materials, systems and methods

We require information from authors about some types of materials, experimental systems and methods used in many studies. Here, indicate whether each material, system or method listed is relevant to your study. If you are not sure if a list item applies to your research, read the appropriate section before selecting a response.

Materials & experimental systems

n/a	Involved in the study	
☒	☐	Antibodies
☒	☐	Eukaryotic cell lines
☒	☐	Palaeontology
☒	☐	Animals and other organisms
☒	☐	Human research participants
☒	☐	Clinical data

Methods

n/a	Involved in the study	
☒	☐	ChIP-seq
☒	☐	Flow cytometry
☒	☐	MRI-based neuroimaging
